# Reel syndrome—An uncommon etiology of ICD dysfunction

**DOI:** 10.1002/ccr3.2682

**Published:** 2020-01-30

**Authors:** George Bazoukis, George Kollias, Gary Tse, Athanasios Saplaouras, Panagiotis Mililis, Antigoni Sakellaropoulou, Angelos‐Michail Kolokathis, Dimitrios Asvestas, Michael Efremidis, Antonios Sideris, Konstantinos P. Letsas

**Affiliations:** ^1^ Department of Cardiology, Electrophysiology Laboratory Evangelismos General Hospital of Athens Athens Greece; ^2^ Department of Medicine and Therapeutics Faculty of Medicine Chinese University of Hong Kong Hong Kong, SAR China; ^3^ Faculty of Medicine Li Ka Shing Institute of Health Sciences Chinese University of Hong Kong Hong Kong, SAR China

**Keywords:** ICD complications, implantable device, Reel syndrome

## Abstract

Reel syndrome occurs due to the rotation of the implantable device on its transverse axis with subsequent coiling of the leads around the pulse generator. Device interrogation and chest X‐ray should be performed in any case of device malfunction.

## Graphical abstract

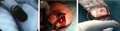

## 1 CLINICAL QUESTION?

What is the reason of ICD dysfunction in that patient?

## 2 ANSWER DISCUSSION

**Figure 1:**
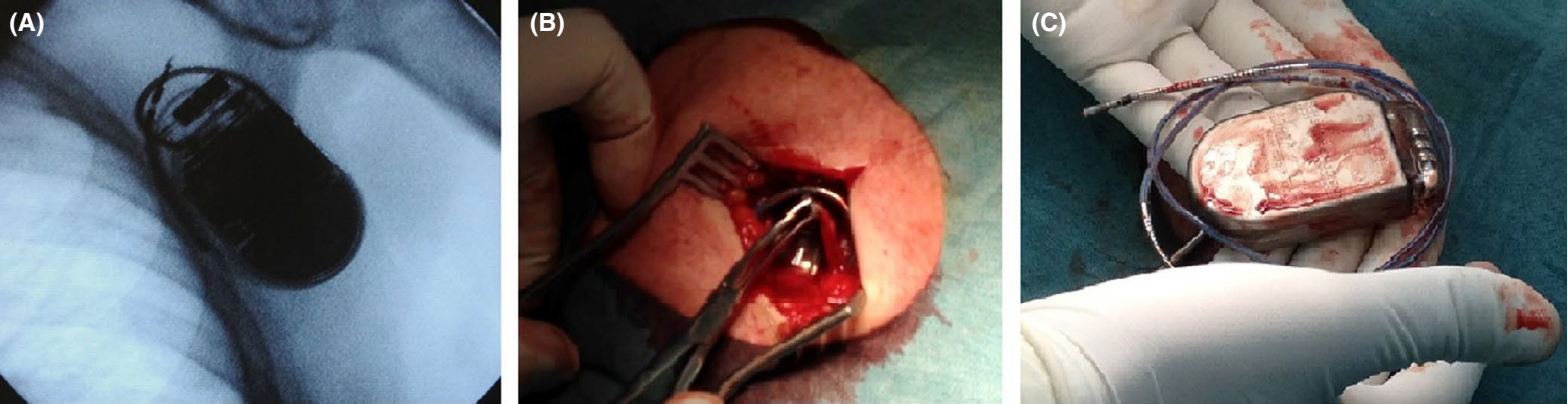
A, Fluoroscopy image showing that the electrode was wound completely around the device. B, C, Surgical views showing the rotation of the generator on its long axis, with subsequent coiling of lead

A 69‐year‐old obese man had undergone implantation of a single‐chamber implantable cardioverter‐defibrillator (ICD) for secondary prevention of sudden cardiac death. An active fixation transvenous electrode (Durata 7120Q, St. Jude Medical) was inserted via the left subclavian vein and placed at the right ventricular apex. A nonabsorbable suture (2/0 Silk) was used for lead fixation with a looping knot around the sleeve. The lead was connected to the ICD device (Fortify ST, St. Jude Medical) implanted subcutaneously in the left subclavian area. A postprocedural X‐ray showed adequate positioning of the lead, and the patient did well and was discharged uneventfully. Six months after the implantation, the patient presented in our clinic for a follow‐up visit complaining about a rhythmic left arm twitching one month ago. The device interrogation revealed failure to sense, failure to capture, and impedance out of range. Chest X‐ray showed coiling of the pacemaker leads around the pulse generator. The patient denied external manipulation of the implanted device but confirmed having performed heavy manual labor during the last months. The patient was consequently admitted to our hospital, and the ICD pocket was opened, the device was extracted, and the patient's rhythmic left arm twitching disappeared the instant the device was explanted. During the procedure and after the device extraction, it became apparent that the electrode was wound completely around the device (Figure 1). The patient was diagnosed with reel syndrome that was first described in 1999.^1^ The lead was uncoiled, and no signs of insulation break or other lead damage could be evident; however, the active fixation spiral could not be retracted within the electrode. Therefore, a new active fixation transvenous electrode (Durata 7120Q, St. Jude Medical) was implanted in the right ventricular apex and connected to the same ICD device. The device was then implanted in the same pocket after it was reduced in size, with additional tight fixation of both the electrode and the ICD device to the muscle fascia. The postreimplantation period was uneventful during a 1‐year follow‐up.

## CONFLICT OF INTEREST

None declared.

## AUTHOR CONTRIBUTIONS

GB: involved in management of the patient, reviewed the literature, wrote the first draft, and approved the final version of the manuscript; GK, AS, PM, AS, AK, DA, ME, and KPL: involved in management of patient, critically reviewed the manuscript, and approved the final version of the manuscript; and GT: critically reviewed and approved the final version of the manuscript.
